# Clinical safety of total glucosides of paeony adjuvant therapy for rheumatoid arthritis treatment: a systematic review and meta-analysis

**DOI:** 10.1186/s12906-021-03252-y

**Published:** 2021-03-26

**Authors:** Bin Liu, Xiang Meng, Yanfang Ma, Huizhen Li, Yuqi Liu, Nannan Shi, Yaolong Chen, Yanping Wang, Cheng Lu

**Affiliations:** 1 Institute of Basic Research in Clinical Medicine, China Academy of Chinese Medical Sciences, Beijing, China; 2 Evidence-Based Medicine Center, School of Basic Medical Sciences, Lanzhou University, Lanzhou, China; 3 WHO Collaborating Centre for Guideline Implementation and Knowledge Translation, Lanzhou University, Lanzhou, China; 4 Chinese GRADE Center, Lanzhou, China

**Keywords:** Rheumatoid arthritis, Total glucosides of paeony, Adjuvant therapy, Clinical safety, Systematic review, Meta-analysis

## Abstract

**Background:**

Total glucosides of paeony (TGP), an active compound extracted from the roots of *Paeonia lactiflora Pallas*, has been increasingly used as the adjunctive therapy for rheumatoid arthritis (RA) patients. Though TGP could mitigate the unanticipated adverse effects during the conventional treatment of RA, high-quality evidence-based meta-analysis data on this subject are still insufficient. The objective of this study is to evaluate the clinical safety of TGP adjuvant therapy in the RA treatment.

**Methods:**

PubMed, EMBASE, Web of Science, China Network Knowledge Infrastructure (CNKI), SinoMed and WanFang Data were retrieved for randomized controlled trials (RCTs) and cohort study about TGP adjuvant therapy in patients with RA up to 28 January 2021. Literatures with eligibility criteria and information were screened and extracted by two researchers independently. The RevMan5.3 software was used for data analysis with effect estimates as risk ratio (RR) with 95% confidence interval (CI).

**Results:**

A total of 39 studies involving 3680 RA participants were included. There were 8 comparisons: TGP plus methotrexate (MTX) therapy versus MTX therapy, TGP plus leflunomide (LEF) therapy versus LEF therapy, TGP plus MTX and LEF therapy versus MTX plus LEF therapy, TGP plus tripterygium glycosides (TG) therapy versus TG therapy, TGP plus meloxicam (MLX) therapy versus MLX therapy and TGP plus sulfasalazine (SSZ) therapy versus SSZ therapy, TGP plus iguratimod (IGU) therapy versus IGU therapy, TGP plus prednisone acetate tablets (PAT) therapy versus PAT therapy. The meta-analysis results showed that the occurrence of hepatic adverse effect (RR = 0.31, 95% CI = 0.23–0.41, *P* < 0.00001) and leukopenia (RR = 0.41, 95% CI = 0.26–0.66, *P* = 0.0002) in TGP adjuvant therapy was significant decreased compared with non-TGP therapy. However, only TGP plus LEF therapy (RR = 0.22, 95% CI = 0.08–0.60, *P* = 0.003) and TGP plus MTX and LEF therapy (RR = 0.31, 95% CI = 0.22–0.42, *P* < 0.00001) had statistical difference in the subgroups of hepatic adverse effect. In leukopenia, TGP plus MTX and LEF therapy (RR = 0.47, 95% CI = 0.25–0.87, *P* = 0.02) had statistical difference.

**Conclusions:**

This meta-analysis indicated that TGP adjuvant therapy might alleviate the incidence of hepatic adverse effect and leukopenia for the RA treatment compared to non-TGP therapy. The clinical safety of TGP adjuvant therapy warrant further investigation in experimental studies.

**Supplementary Information:**

The online version contains supplementary material available at 10.1186/s12906-021-03252-y.

## Background

Rheumatoid arthritis (RA) is a chronic autoimmune disease with an increasing global prevalence, characterized by chronic synovial inflammation, cartilage and joint erosion, pannus formation, joint abnormalities and ankylosis [1]. At present, four types of drugs including non-steroidal anti-inflammatory drugs, disease-modifying anti-rheumatic drugs, glucocorticoid and biological agents are commonly used for the RA treatment [2, 3]. However, some unanticipated adverse effects, such as gastrointestinal effects, skin effects, hepatotoxicity, and neurological symptoms, occur occasionally during the long therapeutic procedure [4–7]. In clinical practice, adjuvant drug treatments have been often applied to reduce adverse effects [8] and many patients are also willing to approve such adjuvant therapy [9].

In classical Chinese herbal textbooks and the Pharmacopoeia of China, *Paeonia lactiflora Pallas* is often referred to as a holy drug to protect the liver [10]. Traditional Chinese medicine posits that the liver holds blood, and modern medical research has confirmed that the liver is not only an important metabolic organ, but also an immune organ. The liver is rich in natural immune cells, and its unique immune-cell composition plays an important role in the formation of its functional characteristics [11, 12]. As an active compound extracted from the roots of *Paeonia lactiflora Pallas*, total glucosides of paeony (TGP) has been increasingly used as the adjunctive therapy for RA patients [13]. TPG contains more than fifteen monoterpene glycosides, such as paeoniflorin, albiflorin, oxypaeoniflorin, benzoylpaeoniflorin, benzoyloxypeoniflorin [14]. With the development of chemical component separation technology and analysis technology, more and more new monoterpene glycoside components in TGP are separated and analyzed. While TGP has good anti-inflammatory and immunosuppressive effects [15, 16], it also has been confirmed to be hepatoprotective [17]. In the meantime, TGP combined with methotrexate (MTX) and leflunomide (LEF) might be more effective against RA, showing a reduced erythrocyte sedimentation rate, C-reactive protein level and rheumatoid factor. Besides, hepatotoxicity has also been decreased significantly in the TGP adjuvant therapy [18]. Though TGP could mitigate the unanticipated hepatotoxicity during the conventional treatment of RA, high-quality evidence-based meta-analysis data on clinical safety of TGP adjuvant therapy are still insufficient. This study aims to evaluate the clinical safety of TGP adjuvant therapy in the RA treatment.

## Methods

### Protocol and registration

This meta-analysis had been registered on PROSPERO of the Centre for Reviews and Dissemination (NO: CRD42018118519).

### Eligibility and exclusion criteria

Eligibility criteria were as following: (1) participants were diagnosed with RA based on the criteria revised by the American College of Rheumatology in 1987 or the ACR/European League Against Rheumatism in 2010; (2) The trial was claimed to be a randomized controlled trial (RCT) or cohort study comparing the TGP adjuvant therapy to non-TGP therapy; (3) the language of studies was limited to English or Chinese; (4) predefined outcome: clinical adverse effect, including gastrointestinal adverse effect, cutaneous adverse effect, hepatic adverse effect, leukopenia and nervous system adverse effect based on the report of National Medical Products Administration, literatures and clinical physicians’ opinion.

Exclusion criteria were as following: (1) participants in studies with other serious diseases; (2) reviews and trials published only as abstracts; (3) there are additional treatment factors in the control group and/or the combination group; (4) the language was not written in English or Chinese.

### Search strategy

To assess the clinical safety of TGP adjuvant therapy for RA, we searched 6 databases including PubMed (1966–2021), EMBASE (1974–2021), Web of Science (1980–2021), China Network Knowledge Infrastructure (CNKI) (1979–2021), SinoMed (1978–2021) and WanFang Data (1997–2021) from inception to 28 January 2021. The search strategies were as follows: “Rheumatoid Arthritis” OR “Caplan Syndrome” OR “Rheumatoid Nodule” OR “Rheumatoid Vasculitis” OR “Sjogren’s Syndrome” OR “Adult-Onset Still’s Disease” AND “total glucosides of paeonia” OR “total glucosides of paeony” OR “bai shao zong gan” AND “Adverse Reaction” OR “Toxicity” OR “Side Effect” OR “Toxic Reaction”. Various combinations of the keywords were applied and the search strategy amalgamated MeSH terms with text words search. The detailed search strategy for each electronic database is available in Additional file 1.

### Study selection and data extractions

The titles and abstracts of the searched results were assessed by two authors independently. Then the full texts of potentially eligible studies were screened to identify the final included studies. The standard form was pre-designed for this systematic review. The extracted data were as follows: study characteristics (authors, title, etc.), methodological information (randomization, allocation concealment, blinding, and follow-up, selective outcome reporting), patient characteristics (number of patients, age, gender, etc.), intervention, control, and outcomes. Any disagreements were discussed or sought a third opinion.

### Assessment of risk of bias in included studies

Two authors used the tools developed by Higgins and Green in the Cochrane Systematic Review Intervention Handbook to assess the risk of bias in inclusion in studies independently. The following risk of bias: selection bias (random sequence generation and allocation concealment), performance bias (blinding of participants and personnel), detection bias (blindness of researchers performing outcome evaluation), attrition bias (incomplete outcome data), reporting bias (selective reporting) and other sources of bias were assessed. Meanwhile, “low risk”, “high risk” or “unclear risk” judgment had been provided for each aspect of the risk of bias.

### Statistical analysis

Data were aggregated using risk ratio (RR) with 95% confidence intervals (CI) for binary outcomes. Revman 5.3 Software was used from the Cochrane Collaboration for data analyses. Meta-analysis was performed if the trials had a good homogeneity on study design, participants, interventions, control, and outcome. Statistical heterogeneity was tested by examining both the Chi-squared test and the Isquared statistic (*I*^*2*^), which means that an *I*^*2*^ is greater than 50% and *P* is less than or equal to 0.1 indicating the possibility of statistical heterogeneity and employing random-effects model. If there was no statistically significant heterogeneity between studies in the meta-analysis, the fixed effect model was adopted. Funnel plots and Egger’s test were used to assess the publication bias if more than 10 studies tested the same outcome in one meta-analysis.

## Results

### Search results

**Fig. 1:**
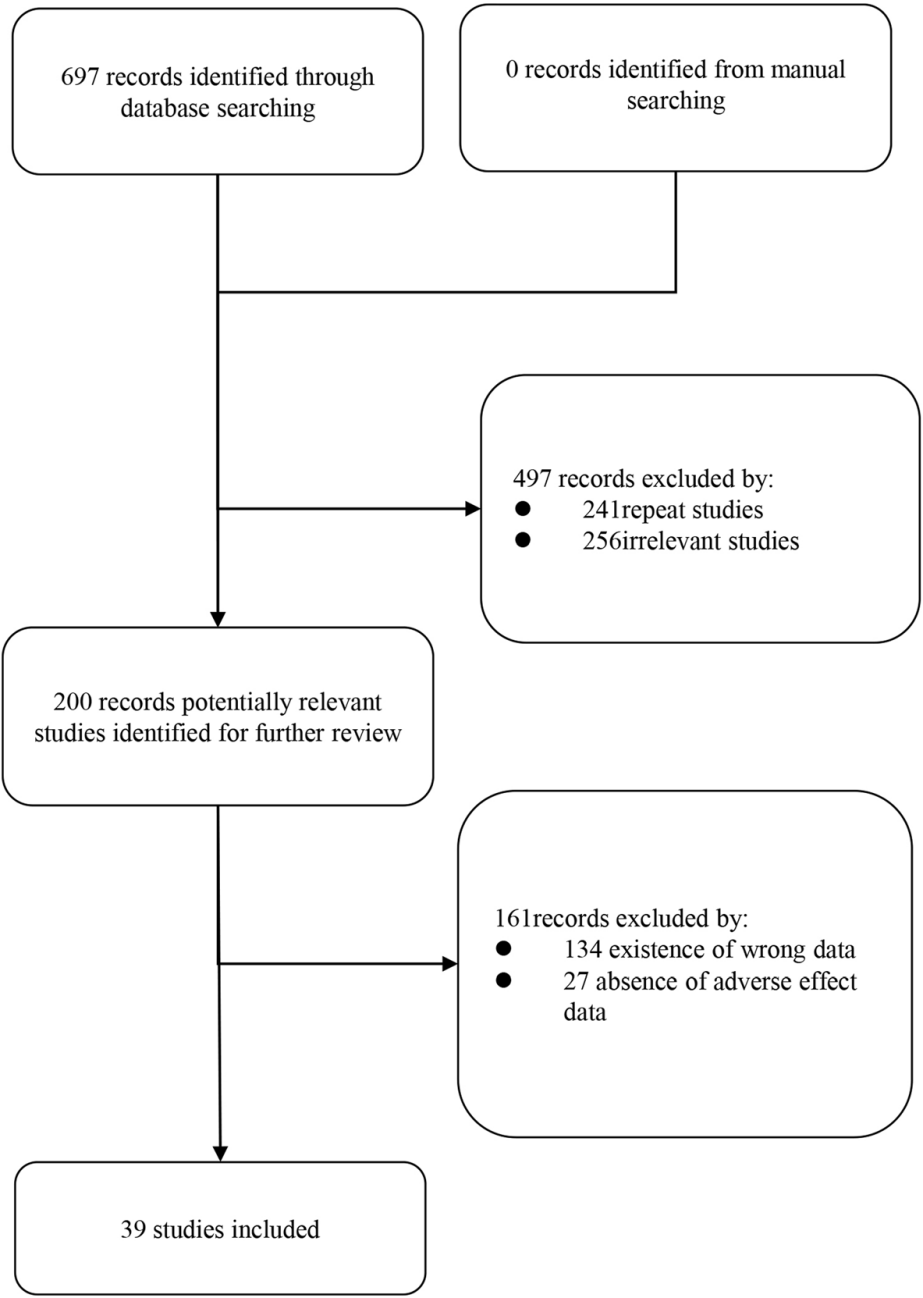
Flow diagram illustrating the process of identifying articles for selection

There were 697 articles identified by literature search: SinoMed (*n* = 184), CNKI (*n* = 127), WanFang Data (*n* = 310), PubMed (*n* = 24), Web of Science (*n* = 32), EMBASE (*n* = 20). NoteExpress was used to conduct duplicate checking and ultimately selected 200 papers. After reading through the full article, existence of wrong data (*n* = 134) and absence of adverse effect data (*n* = 27) were excluded. In the end, 39 articles [19–57] in total were included for the meta-analysis (Fig. 1). We also tried to get unpublished studies from databases of relevant clinical trials, however, there were no eligible studies.

**Fig. 2:**
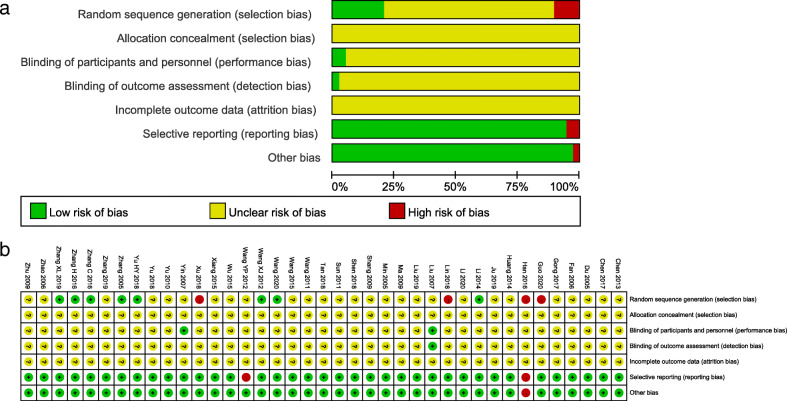
Risk of bias summary (**a**) and Risk of bias graph (**b**)

### Study characteristics

These 39 included trials, which involved 3680 RA participants, were all conducted in mainland China. They were published from 2005 to 2020. There were 1874 and 1806 patients were enrolled in the TGP adjuvant therapy groups and the non-TGP therapy groups, respectively. There were 8 comparisons: TGP plus methotrexate (MTX) therapy versus MTX therapy [20, 21, 31–33, 43, 45, 48, 50–53, 55, 56], TGP plus leflunomide (LEF) therapy versus LEF therapy [34, 37, 40, 47, 49, 54], TGP plus MTX and LEF therapy versus MTX plus LEF therapy [22–29, 35, 38, 39, 42, 44, 46], TGP plus tripterygium glycosides (TG) therapy versus TG therapy [57], TGP plus meloxicam (MLX) therapy versus MLX therapy [36], TGP plus sulfasalazine (SSZ) therapy versus SSZ therapy [41], TGP plus iguratimod (IGU) therapy versus IGU therapy [30] and TGP plus prednisone acetate tablets (PAT) therapy versus PAT therapy [19]. The detailed characteristics are presented in Table 1.

**Table 1:** Characteristics of included studies

Study ID	Patients	Treatment					Control					Course of treatment
		Intervention	N	M/F	Age (Mean ± SD)	Duration of disease (Years)	Intervention	N	M/F	Age (Mean ± SD)	Duration of disease (Years)	
Lin 2016	Patients with RA	MTX + TGP	50	20/30	43.9 ± 5.1	3.5 ± 0.5	MTX	50	20/30	43.7 ± 5.2	3.3 ± 0.6	24 weeks
Yin 2007	Patients with RA	MTX + TGP	30	–	–	–	MTX	30	–	–	–	12 weeks
Gong 2017	Patients with RA	MTX + TGP	40	15/25	67.2 ± 5.2	1.3	MTX	40	17/23	68.8 ± 5.8	1.3	3 months
Chen 2017	Patients with RA	MTX + TGP	40	18/22	32.8 ± 4.9	2.2 ± 0.2	MTX	40	21/19	32.4 ± 5.3	2.2 ± 0.2	24 weeks
Fan 2006	Patients with EORA	MTX + TGP	34	8/26	–	–	MTX	32	7/25	–	–	24 weeks
Min 2005	Patients with RA	MTX + TGP	117	24/93	46.0 ± 13.0	2.4 ± 4.3	MTX	52	17/35	44.0 ± 14.0	2.0 ± 3.3	48 weeks
Du 2005	Patients with RA	MTX + TGP	31	20/11	40.0 ± 6.4	6.8 ± 3.6	MTX	30	18/12	38.0 ± 7.8	4.5 ± 3.2	12 weeks
Sun 2011	Patients with JRA	MTX + TGP	26	12/14	7.6	–	MTX	25	12/13	8.0		26 weeks
Shang 2009	Patients with RA	MTX + TGP	31	11/20	40.0 ± 6.0	4.0 ± 3.0	MTX	28	9/19	39.0 ± 6.0	4.0 ± 3.0	12 weeks
Wang 2012	Patients with JRA	MTX + TGP	31	20/11	10.9 ± 3.3	1.9 ± 1.2	MTX	30	18/12	10.2 ± 2.4	1.8 ± 1.3	12 weeks
Liu 2007	Patients with RA	MTX + TGP	46	–	–	–	MTX	44	–	–	–	24 weeks
Wang 2020	Patients with RA	MTX + TGP	36	16/20	42.8 ± 11.4	13 ± 2.5	MTX	46	17/29	42.8 ± 11.4	12 ± 2	24 weeks
Guo 2020	Patients with RA	MTX + TGP	43	16/27	51.58 ± 3.1	1–14	MTX	43	17/26	51.02 ± 3.16	1-12	12 weeks
Zhu 2009	Patients with RA	MTX + TGP	23	6/17	46 ± 12	–	MTX	23	7/16	47 ± 11	–	24 weeks
Ma 2009	Patients with RA	LEF + TGP	40	–	–	–	LEF	40	–	–	–	12 weeks
Yu 2010	Patients with RA	LEF + TGP	39	11/28	39.0 ± 11.0	1.8 ± 0.8	LEF	40	9/31	46.0 ± 10.0	2.1 ± 0.9	24 weeks
Li 2014	Patients with EORA	LEF + TGP	48	18/30	59.9 ± 6.1	3.9 ± 2.1	LEF	48	20/28	60.2 ± 5.8	4.1 ± 1.9	–
Han 2016	Patients with EORA	LEF + TGP	42	18/24	5.8 ± 7.2	5.36 ± 2.1	LEF	42	12/30	66.2 ± 7.4	5.5 ± 2.3	24 weeks
Wu 2015	Patients with RA	LEF + TGP	50	10/40	–	–	LEF	50	8/42	–	–	12 weeks
Zhao 2006	Patients with RA	LEF + TGP	40	18/22	31.0 ± 8.9	4.0 ± 3.8	LEF	40	14/26	30.0 ± 9.6	5.0 ± 4.9	12 weeks
Zhang 2016	Patients with RA	MTX + LEF + TGP	43	31/12	45.5 ± 4.4	6.3 ± 2.5	MTX + LEF	43	29/14	45.7 ± 4.2	6.1 ± 2.4	3 months
Yu 2018	Patients with RA	MTX + LEF + TGP	42	15/27	58.6 ± 7.5	5.4 ± 1.6	MTX + LEF	38	13/25	58.4 ± 7.5	5.4 ± 1.6	4 weeks
Wang 2011	Patients with RA	MTX + LEF + TGP	33	–	–	–	MTX + LEF	31	–	–	–	12 weeks
Wang YP 2012	Patients with RA	MTX + LEF + TGP	33	–	–	–	MTX + LEF	31	–	–	–	16 weeks
Wang 2015	Patients with RA	MTX + LEF + TGP	40	–	–	–	MTX + LEF	40	–	–	–	12 weeks
Chen 2013	Patients with RA	MTX + LEF + TGP	105	–	–	–	MTX + LEF	89	–	–	–	24 weeks
Xiang 2015	Patients with RA	MTX + LEF + TGP	132	26/106	49.6 ± 12.6	2.2	MTX + LEF	136	39/97	47.6 ± 11.7	2	12 weeks
Tan 2018	Patients with RA	MTX + LEF + TGP	56	19/28	58.4 ± 4.6	8.9 ± 4.3	MTX + LEF	52	20/25	56.8 ± 5.1	10.1 ± 4.5	24 weeks
Xu 2018	Patients with RA	MTX + LEF + TGP	45	15/30	53.46 ± 3.45	5.84 ± 0.35	MTX + LEF	45	16/29	53.76 ± 3.13	5.22 ± 0.28	12 weeks
Yu HY 2018	Patients with RA	MTX + LEF + TGP	40	24/16	47.18 ± 6.92	10.8 ± 3.6	MTX + LEF	40	23/17	47.33 ± 6.67	11.2 ± 3.5	36 weeks
Ju 2019	Patients with RA	MTX + LEF + TGP	60	21/39	40.12 ± 4.37	4.59 ± 0.53	MTX + LEF	60	23/37	39.81 ± 4.42	4.63 ± 0.46	24 weeks
Zhang 2019	Patients with RA	MTX + LEF + TGP	50	28/22	59.62 ± 6.82	5.38 ± 1.56	MTX + LEF	50	30/20	59.71 ± 6.75	5.91 ± 1.42	12 weeks
Liu 2019	Patients with RA	MTX + LEF + TGP	49	25/24	51.62 ± 3.47	2.95 ± 0.64	MTX + LEF	49	26/23	51.75 ± 3.26	2.97 ± 0.58	12 weeks
Zhang XL 2019	Patients with RA	MTX + LEF + TGP	135	41/94	43.12 ± 9.56	–	MTX + LEF	133	40/93	43.12 ± 9.56	–	12 weeks
Zhang 2005	Patients with RA	TG + TGP	30	3/27	51.4 ± 15.2	4.0 ± 3.5	TG	30	2/28	53.6 ± 13.7	3.2 ± 3.2	3 months
Zhang C 2016	Patients with RA	MLX + TGP	49	25/24	57.0 ± 7.5	–	MLX	41	21/20	56.0 ± 6.5	–	12 weeks
Huang 2014	Patients with RA	SSZ + TGP	30	–	–	–	SSZ	60	–	–	–	8 weeks
Shen 2018	Patients with RA	IGU + TGP	30	–	–	–	IGU	30	–	–	–	12 weeks
Li 2020	Patients with RA	PAT+TGP	35	21/14	51.40 ± 4.76	18.67 ± 5.12	PAT	35	15/20	51.52 ± 4.82	18.75 ± 5.16	12 weeks

### Risk of bias of included studies

Thirty-seven trials [19–26, 29–57] were RCTs, 2 trials [27, 28] were cohort study and the baseline characteristics of each trial were comparable. Four trials [21, 28, 33, 34] had a high risk of bias by using the wrong random sequence generation and seven trials [20, 29, 35, 36, 40, 43, 57] had a low risk of bias because of their generating random numbers by using the random number table or centralized randomization. Two trials [51, 52] used blinding of participants and personnel. One trial [52] had a low risk of bias on account of it was double-blind and reported the blinding of outcome assessment. Two trials [34, 44] had a high risk of bias in selective reporting. All trials had an unclear risk of bias allocation concealment and blinding of outcome assessment because of incomplete outcome data (Fig. 2).

### Effects of interventions

**Fig. 3:**
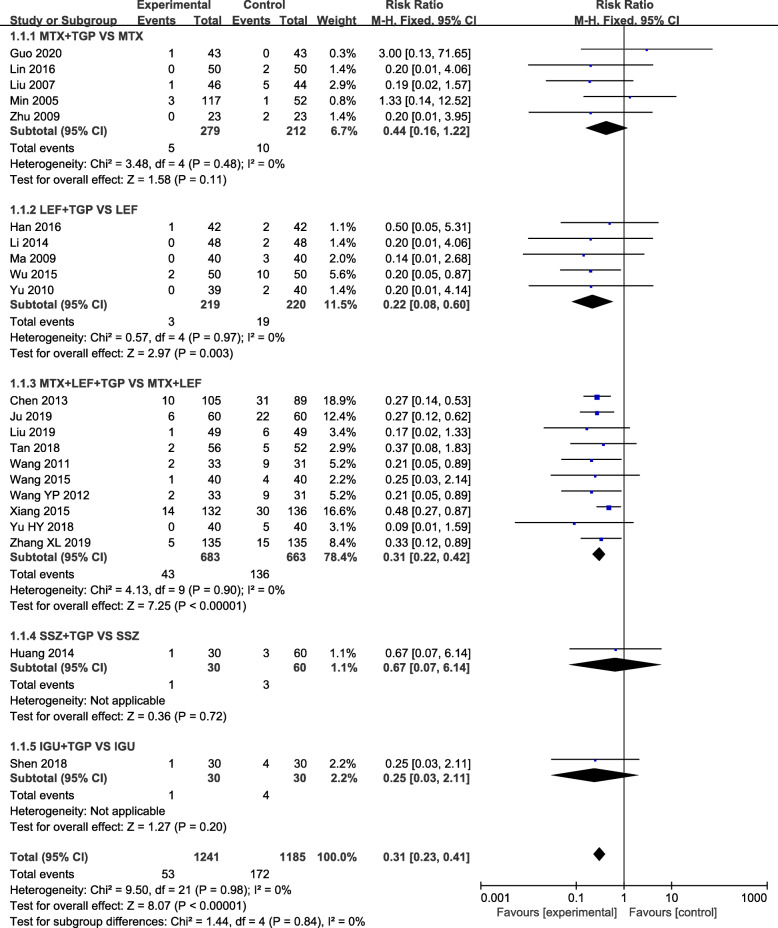
Forest plots for hepatic adverse effect of TGP adjuvant therapy versus non-TGP therapy

**Fig. 4:**
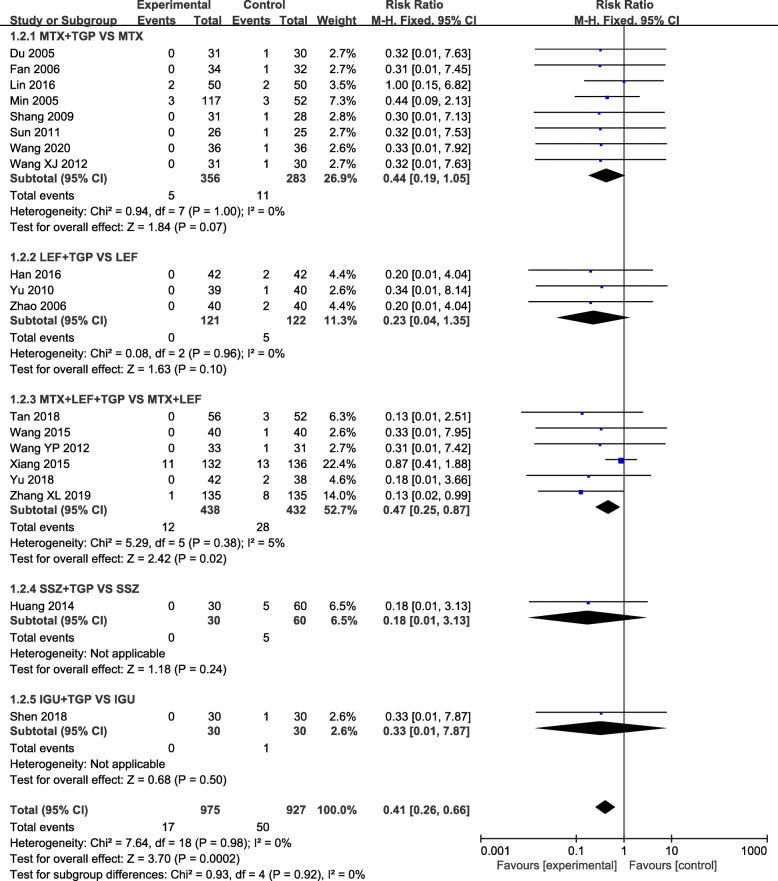
Forest plots for leukopenia of TGP adjuvant therapy versus non-TGP therapy

**Fig. 5:**
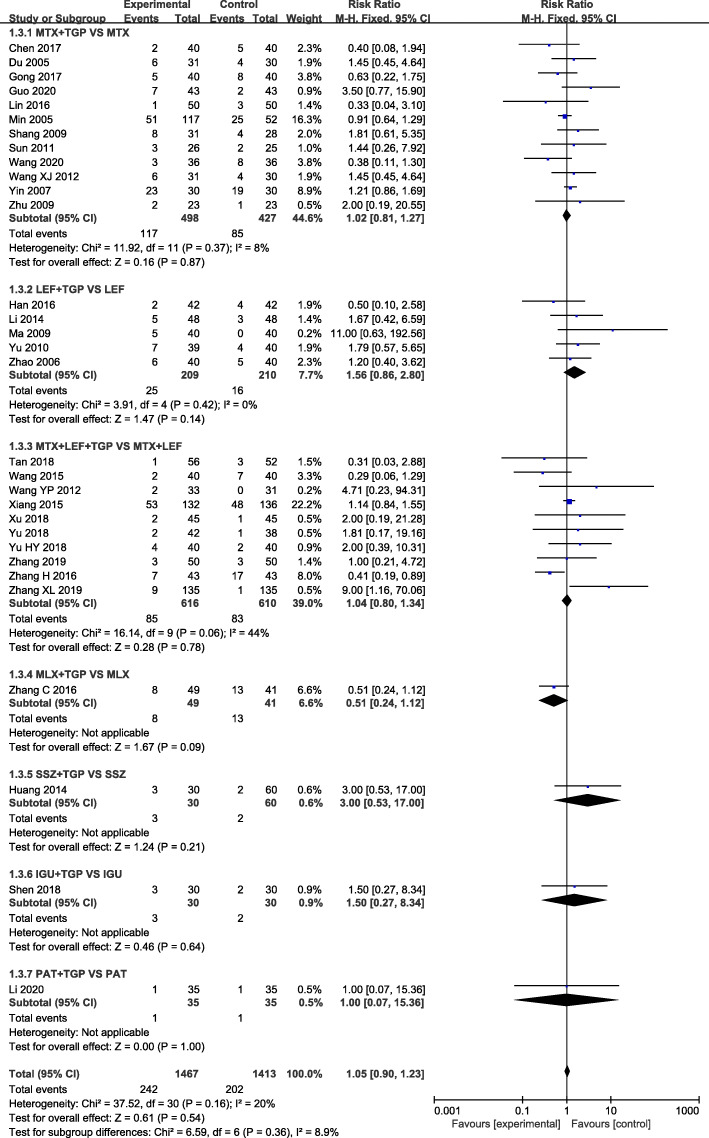
Forest plots for gastrointestinal adverse effect of TGP adjuvant therapy versus non-TGP therapy

**Fig. 6:**
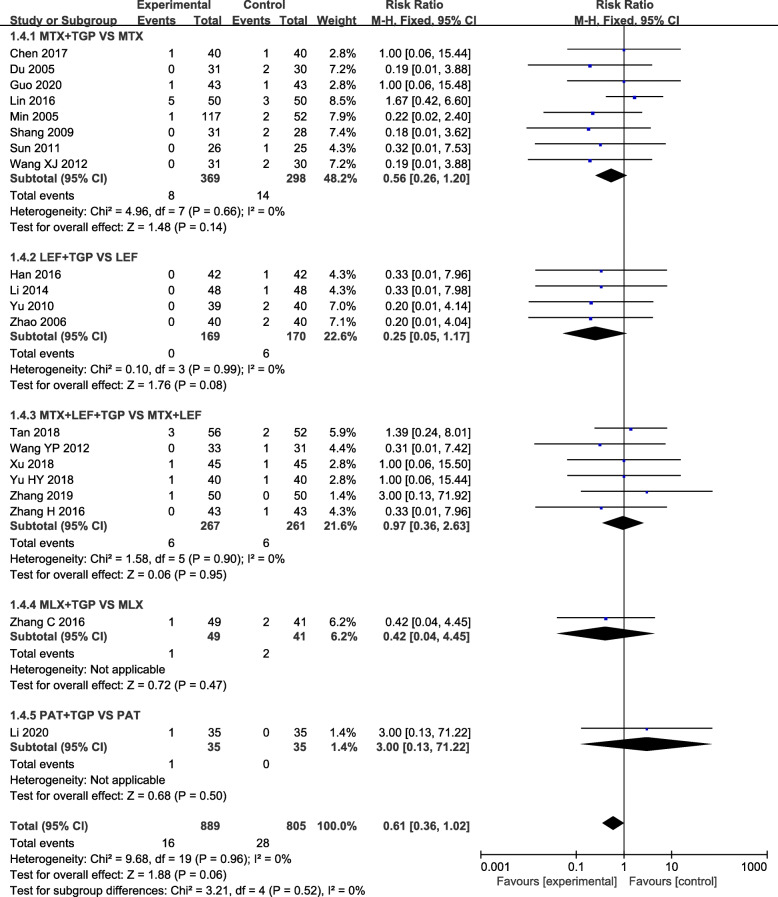
Forest plots for cutaneous adverse effect of TGP adjuvant therapy versus non-TGP therapy

**Fig. 7:**
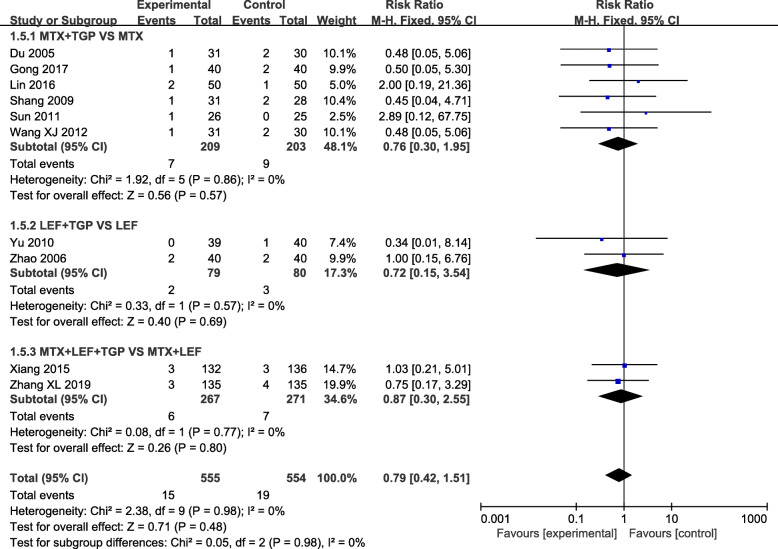
Forest plots for nervous system adverse effect of TGP adjuvant therapy versus non-TGP therapy

Our analysis revealed that TGP adjuvant therapy could decrease the incidence of hepatic adverse effect (RR = 0.31, 95% CI = 0.23–0.41, *P* < 0.00001) (Fig. 3) and leukopenia (RR = 0.41, 95% CI = 0.26–0.66, *P* = 0.0002) (Fig. 4) compared with non-TGP therapy. However, there were no statistical difference of gastrointestinal adverse effect (RR = 1.05, 95% CI = 0.90–1.23, *P* = 0.54) (Fig. 5), cutaneous adverse effect (RR = 0.61, 95% CI = 0.36–1.02, *P* = 0.06) (Fig. 6) and nervous system adverse effect (RR = 0.79, 95% CI = 0.42–1.15, *P* = 0.48) (Fig. 7) between TGP adjuvant therapy and non-TGP therapy.

22 studies evaluated the hepatic adverse effect with 1241 patients in the TGP adjuvant therapy group and 1185 patients in the non-TGP therapy group. Although TGP adjuvant therapy can decrease the incidence of hepatic adverse effect, only TGP plus LEF therapy (RR = 0.22, 95% CI = 0.08–0.60, *P* = 0.003) and TGP plus MTX and LEF therapy (RR = 0.31, 95% CI = 0.22–0.42, *P* < 0.00001) had statistical difference in the subgroups. Meanwhile, no statistical difference was found in the following four comparisons of gastrointestinal adverse effect, nervous system adverse effect and cutaneous adverse effect: (1) TGP plus MTX versus MTX; (2) TGP plus LEF versus LEF; (3) TGP plus MTX and LEF versus MTX plus LEF; (4) TGP plus MLX/SSZ/IGU/PAT versus MLX/SSZ/IGU/PAT.

### Sensitivity analysis

Sensitivity analysis was implemented to evaluate the results and we found that there was no significant change, suggesting that the sensitivity was low and the results were more stable.

## Discussion

This systematic review aims to assess the clinical safety of TGP adjuvant therapy in the RA treatment. 39 trials involving 3681 RA patients were included in this review with language restrictions in both Chinese and English. Clinical adverse effect including gastrointestinal adverse effect, cutaneous adverse effect, hepatic adverse effect, leukopenia, and nervous system adverse effect were analyzed in TGP adjuvant therapy and non-TGP therapy. The meta-analysis results signified that the occurrence of hepatic adverse effect and leukopenia in TGP adjuvant therapy was significant decreased. However, no statistical significance was found in subgroup analysis of gastrointestinal adverse effect, nervous system adverse effect and cutaneous adverse effect, the reason for this might be the sample size was small and the quality of methodologies used in the included studies was poor.

Hepatic adverse effect is one of the main adverse effects of MTX and LEF in the treatment of RA. Elevated levels of alanine transaminase (ALT) and aspartate transaminase (AST) in serum suggest the architecture of hepatic cell may be injured. Although the reversibility of mild liver enzyme elevations in a clinical trial setting is reassuring, the potential for increased hepatic toxicity with the use of MTX and LEF combination should be recognized [58, 59]. Previous study demonstrated that TGP could reduce the activity of ALT and AST in liver injury and the content of MDA in liver homogenate, increase the activity of SOD and protect drug-induced liver injury [60]. Meanwhile, TGP might protect hepatocytes through modulating oxidative damage improving the changes in liver structure and alleviating lobular necrosis in an acute liver injury rat model. In addition to, the increased expression of iNOS and CYP2E1 in liver of acute liver injury rat model also had been attenuated by TGP [61]. Those may be the reasons why TGP plus LEF (and MTX) therapy decreased the incidence of hepatic adverse effect compared to LEF (and MTX) therapy, but it requires further research and definite evidence.

In this meta-analysis, TGP adjuvant therapy can decrease the incidence of leukopenia, but no statistical difference was found in the subgroups. Meanwhile, TGP is effective and safety in treatment patients of leukopenia with systemic lupus erythematosus [62]. Although there was no statistical significance in gastrointestinal adverse effect, cutaneous adverse effect and nervous system adverse effect, a certain downward trend showed in the incidence of these adverse effects.

This review has several limitations. Firstly, almost all trials had focused on therapeutic effect, and there were a few trials focused on clinical safety evaluation specifically, so the sample of raw data included in this review was small. Although we searched the Chinese and English databases, all the included trials were conducted in China, which may have introduced potential selection bias and limited the external promotion of the evidence. Secondly, the purity, concentrations, quality control and chemical analysis of TGP were not presented in all the original studies. Thirdly, clinical safety may not be sufficient for safety of drug, since the herbal extracts were used together with western medications thus herb-drug interaction should also be considered and evaluated. In fact, we searched the literature about herb-drug interaction of TGP and the other herbal extracts, but literatures is rare, which may also be a limitation of this study. This is something we need to further study in the future. Although the limitations mentioned above may undermine the level of evidence of this meta-analysis, the selected trials are highly comparable, and the documents were selected in strict accordance with inclusion criteria. To some extent, the safety data from these trials also provided us with some research directions.

This review indicates that high quality studies would be warranted to confirm the clinical safety. First, clinical trial protocols should be predetermined based on research questions and registered to ensure that research can be conducted according to predetermined criteria. Meanwhile, factors that may influence or reduce the quality of the research methodology should be controlled during the research process. Appropriate methods are needed to generate random numbers, assign hidden, lose data processing, and avoid performance biases and other biases. In addition, statistics and observations of clinical data are required to unify the outcomes of clinical safety.

## Conclusions

This meta-analysis indicated that TGP adjuvant therapy might alleviate the incidence of hepatic adverse effect and leukopenia for the RA treatment compared to non-TGP therapy. Further large sample, multicenter, high-quality studies are still needed to confirm the clinical safety of TGP adjuvant therapy.

## Supplementary Information

**Additional file 1.** The detailed search strategies.

## Data Availability

All data generated during this study are included in this article and its supplementary information files.

## Abbreviations

TGP: Total glucosides of paeony
RA: Rheumatoid arthritis
RCTs: Randomized controlled trials
RR: Risk ratio
CI: Confidence interval
MTX: Methotrexate
LEF: Leflunomide
TG: Tripterygium glycosides
MLX: Meloxicam
SSZ: Sulfasalazine
ALT: Transaminase
AST: Aspartate transaminase
IGU: Iguratimod
PAT: Prednisone acetate tablets
