# Supplementary material for: Clinical safety of total glucosides of paeony adjuvant therapy for rheumatoid arthritis treatment: a systematic review and meta-analysis
Source: BMC Complement Med Ther. 2021 Mar 26;21:102. doi: 10.1186/s12906-021-03252-y (PMC8004450; doi:10.1186/s12906-021-03252-y)
Supplement: Supplementary file 1 — Additional file 1. The detailed search strategies. [file 12906_2021_3252_MOESM1_ESM.docx]

**Additional file 1** The detailed search strategies

**The search strategy of SinoMed (n=184)**

1. “Arthritis, Rheumatoid” [Unweighted: extension]
2. “Rheumatoid Arthritis” [Common fields: intelligent]
3. “Chronic Infectious Arthritis” [Common fields: intelligent]
4. “Rheumatoid Pneumoconiosis” [Common fields: intelligent]
5. “Caplan Syndrome” [Common fields: intelligent]
6. “Felty Syndrome” [Common fields: intelligent]
7. “Rheumatoid Nodule” [Common fields: intelligent]
8. “Sjogren Syndrome” [Common fields: intelligent]
9. OR/1-8
10. “Paeonia lactiflora Pallas” [Unweighted: extension]
11. “Paeonia lactiflora Pallas” [Common fields: intelligent]
12. “Pavlin” [Common fields: intelligent]
13. “Pa Fu Lin” [Common fields: intelligent]
14. OR/10-13
15. “Adverse Drug Reaction Reporting System” [Unweighted: extension]
16. “Safety” [Common fields: intelligent]
17. “Toxicity” [Common fields: intelligent]
18. “Side Reaction” [Common fields: intelligent]
19. “Side Effect” [Common fields: intelligent]
20. “Adverse Reaction” [Common fields: intelligent]
21. “Toxic Effects” [Common fields: intelligent]
22. “Toxic Reaction” [Common fields: intelligent]
23. “Attenuation” [Common fields: intelligent]
24. OR/15-23
25. #9 AND #14 AND #24

**The search strategy of CNKI (n=127)**

1. Rheumatoid Arthritis [Topic]
2. Chronic Infectious Arthritis [Topic]
3. Rheumatoid Pneumoconiosis [Topic]
4. Caplan Syndrome [Topic]
5. Felty Syndrome [Topic]
6. Rheumatoid Nodule [Topic]
7. Sjogren Syndrome [Topic]
8. OR/1-7
9. “total glucosides of paeony” [Topic]
10. “Pavlin” [Topic]
11. “Pa Fu Lin” [Topic]
12. OR/9-11
13. Attenuation [Topic]
14. Toxicity [Topic]
15. Safety [Topic]
16. Side Reaction [Topic]
17. Side Effect [Topic]
18. Adverse Reactions [Topic]
19. Toxic Effects [Topic]
20. Toxic Reaction [Topic]
21. OR/13-20
22. #8 AND #12 AND #21

**The search strategy of WanFang Data (n=310)**

1. Rheumatoid Arthritis [Topic]
2. Chronic Infectious Arthritis [Topic]
3. Rheumatoid Pneumoconiosis [Topic]
4. Caplan Syndrome [Topic]
5. Felty Syndrome [Topic]
6. Rheumatoid Nodule [Topic]
7. Sjogren Syndrome [Topic]
8. OR/1-7
9. “total glucosides of paeony” [Topic]
10. “Pavlin” [Topic]
11. “Pa Fu Lin” [Topic]
12. OR/9-11
13. Attenuation [Topic]
14. Toxicity [Topic]
15. Safety [Topic]
16. Side Reaction [Topic]
17. Side Effect [Topic]
18. Adverse Reactions [Topic]
19. Toxic Effects [Topic]
20. Toxic Reaction [Topic]
21. OR/13-20
22. #8 AND #12 AND #21

**The search strategy of PubMed (n=24)**

1. "total glucosides of paeonia" [Title/Abstract]
2. "total glucosides of paeony" [Title/Abstract]
3. "bai shao zong gan" [Title/Abstract]
4. "TGP" [Title/Abstract]
5. OR/1-4
6. "Arthritis, Rheumatoid" [MeSH]
7. "Rheumatoid Arthritis" [Title/Abstract]
8. “Caplan Syndrome” [Title/Abstract]
9. “Felty Syndrome” [Title/Abstract]
10. “Rheumatoid Nodule” [Title/Abstract]
11. “Rheumatoid Vasculitis” [Title/Abstract]
12. “Sjogren's Syndrome” [Title/Abstract]
13. “Adult-Onset Still's Disease” [Title/Abstract]
14. OR/6-13
15. "Adverse effects" [Subheading]
16. "Long Term Adverse Effects" [MeSH]
17. "Drug-Related Side Effects and Adverse Reactions" [MeSH]
18. "Patient Harm" [MeSH]
19. "Patient Safety" [MeSH]
20. "Adverse Drug Reaction*" [All Fields]
21. "Adverse Event*" [All Fields]
22. "adverse Effect*" [All Fields]
23. "Adverse Reaction*" [All Fields]
24. "Poisoning" [All Fields]
25. "Toxicity" [All Fields]
26. "Side Reaction*" [All Fields]
27. "Side Effect*" [All Fields]
28. "Toxic Reaction*" [All Fields]
29. "Toxic Effect*" [All Fields]
30. "Secondary Action*" [All Fields]
31. OR/15-30
32. #5 AND #14 AND #31

(((((((((((("Adverse Drug Reaction*") OR "Adverse Event*") OR "adverse Effect*") OR "Adverse Reaction*") OR Poisoning) OR Toxicity) OR "Side Reaction*") OR "Side Effect*") OR "Toxic Reaction*") OR "Toxic Effect*") OR "Secondary Action*")) OR (((("adverse effects" [Subheading] OR "Long Term Adverse Effects"[Mesh]) OR "Drug-Related Side Effects and Adverse Reactions"[Mesh]) OR "Patient Harm"[Mesh]) OR "Patient Safety"[Mesh])

**The search strategy of Web of Sciense (n=32)**

1. "total glucosides of paeonia" [Topic]
2. "total glucosides of paeony" [Topic]
3. "bai shao zong gan" [Topic]
4. "TGP" [Topic]
5. OR/1-4
6. "Rheumatoid Arthritis" [Topic]
7. “Caplan Syndrome” [Topic]
8. “Felty Syndrome” [Topic]
9. “Rheumatoid Nodule” [Topic]
10. “Rheumatoid Vasculitis” [Topic]
11. “Sjogren's Syndrome” [Topic]
12. “Adult-Onset Still's Disease” [Topic]
13. OR/6-12
14. "Adverse effect*" [Topic]
15. "Adverse Reaction*" [Topic]
16. "Patient Harm" [Topic]
17. "Patient Safety" [Topic]
18. "Adverse Drug Reaction*" [Topic]
19. "Adverse Event*" [Topic]
20. "Poisoning" [Topic]
21. "Toxicity" [Topic]
22. "Side Reaction*" [Topic]
23. "Side Effect*" [Topic]
24. "Toxic Reaction*" [Topic]
25. "Toxic Effect*" [Topic]
26. "Secondary Action*" [Topic]
27. OR/14-26
28. #5 AND #13 AND #27

**The search strategy of Embase (n=20)**

1. 'total glucosides of paeonia': ab,ti,kw
2. 'total glucosides of paeony': ab,ti,kw
3. 'bai shao zong gan': ab,ti,kw
4. 'TGP': ab,ti,kw
5. OR/1-4
6. 'rheumatoid arthritis'/exp
7. 'adult onset still disease*': ab,ti,kw
8. 'felty syndrome': ab,ti,kw
9. 'juvenile rheumatoid arthritis': ab,ti,kw
10. 'rheumatoid nodule': ab,ti,kw
11. 'rheumatoid arthritis': ab,kw,ti
12. OR/6-11
13. 'adverse event'/exp
14. 'patient safety'/exp
15. 'adverse drug reaction'/exp
16. 'toxicity and intoxication'/exp
17. 'side effect'/exp
18. 'adverse event': ab,kw,ti
19. 'patient safety': ab,kw,ti
20. 'adverse drug reaction': ab,kw,ti
21. 'side effect*': ab,kw,ti
22. 'adverse effect*': ab,kw,ti
23. 'adverse reaction*': ab,kw,ti
24. 'patient harm': ab,kw,ti
25. 'adverse drug reaction*': ab,kw,ti
26. 'poisoning': ab,kw,ti
27. 'side reaction*': ab,kw,ti
28. 'toxic reaction*': ab,kw,ti
29. 'toxic effect*': ab,kw,ti
30. 'secondary action*': ab,kw,ti
31. 'patient risk': ab,kw,ti
32. OR/13-31
33. #5 AND #12 AND #32
34. #33 [medline]/lim
35. #33 NOT #34
